# Anaphylaxis Following Fish Ingestion in a Military Member

**DOI:** 10.7759/cureus.89066

**Published:** 2025-07-30

**Authors:** Ava P Klein, Jackson L Howell, Christopher Coop

**Affiliations:** 1 Perioperative Flight, Keesler Medical Center, Biloxi, USA; 2 Pediatrics, Keesler Medical Center, Biloxi, USA; 3 Allergy and Immunology, Keesler Medical Center, Biloxi, USA

**Keywords:** active-duty military personnel, allergy and immunology, fish allergy, food-induced anaphylaxis, military deployment, military healthcare, military readiness

## Abstract

We present the case of a 33-year-old active-duty male with a history of anaphylaxis following fish ingestion, allergic rhinoconjunctivitis, and multiple environmental and food allergies confirmed with serum-specific IgE testing. This report discusses the patient’s clinical presentation, diagnostic evaluation, and management plan, including allergen avoidance, epinephrine use, environmental control strategies, and a case of unexpected severe anaphylaxis despite low serum-specific IgE levels. Military service requires stringent physical capabilities, and deployment requirements add an additional layer of complexity to the outlook of this case. These conditions underscore the importance of thoroughly analyzing allergic diseases in military personnel and leveraging clinical data to support informed decisions about their medical readiness. This includes implementing individualized care plans that emphasize strict allergen avoidance, ensuring consistent access to emergency medications, and applying environmental control strategies to reduce exposure risks. Additionally, healthcare providers should account for the unique challenges posed by austere deployment environments, such as limited food options and increased exposure to environmental allergens, when assessing fitness for duty. This particular service member was deemed deployable but ultimately, a comprehensive, data-driven approach is essential for balancing the health needs of service members with the operational demands of military deployment.

## Introduction

Food allergy affects up to 10% of the U.S. population and is one of the most common causes of anaphylaxis, especially in young adults [1]. Fish and shellfish are among the most frequently indicated allergens in adult cases of anaphylaxis [2]. Serum specific IgE testing is one of the diagnostic tests used to help verify reactivity to an allergen. For cod, a result of 20 kUA/l is indicative of a severe allergic sensitivity. Lower IgE test results are generally associated with less severe sensitivities [3]. Seasonal allergic rhinoconjunctivitis, typically caused by pollen from grasses, weeds, and trees, affects over 20-40% of adults and can significantly impair quality of life and occupational functioning. Seasonal allergic rhinoconjunctivitis can also be exacerbated by other environmental factors such as dust, dirt particles, and severe air pollution [4]. These conditions are traditionally managed through medication therapies and avoidance of allergens. However, military members are exposed to conditions where traditional health safeguards are not available. Additionally, military members are exposed to rigorous environmental conditions that may aggravate otherwise manageable environmental allergies. Supply chain issues in overseas locations might also diminish the availability of potentially life-saving medications for individuals suffering from allergic diseases. In military populations, timely recognition and management of allergic diseases are essential to preserving operational readiness and ensuring safety in higher-risk environments [5]. In this report, we discuss the case of anaphylaxis induced by food allergy in an active-duty member, reflecting the possibility for enhanced risk from allergic diseases and the implications it has on medical readiness and deployment safety.

## Case presentation

A 33-year-old male active-duty U.S. Air Force member presented for evaluation of suspected food and environmental allergies. He described an acute episode of periocular and perioral edema, urticaria, throat tightness, and dyspnea shortly after accidentally consuming cod, for which he received emergency treatment with intramuscular epinephrine 0.3 mg, diphenhydramine 50 mg intravenous (IV), and methylprednisolone 125 mg IV. He reported a previous occurrence of similar oral pruritus and swelling after shellfish ingestion and had since avoided all seafood per the direction of his allergist. This previous occurrence happened six months prior to the cod reaction, and it was following shrimp ingestion. He self-treated with over-the-counter antihistamines and received follow-up treatment by an allergist where he was prescribed an epinephrine auto-injector, demonstrating proper use in case of exposure. The patient also reported seasonal allergic symptoms consistent with allergic rhinoconjunctivitis, including nasal congestion, sneezing, watery and itchy eyes, and postnasal drip, primarily during the spring and summer. He managed the seasonal allergic symptoms by using over-the-counter allergy medications as needed. His medical history included major depressive disorder, chronic PTSD, generalized anxiety disorder, and alcohol use disorder (in remission). He denied asthma, latex, or venom allergies. The patient self-reported medication allergies to penicillin and sulfa drugs, previously associated with urticaria and rash, but these allergies were not confirmed by any invasive testing. He lived in a carpeted residence with no pets or visible mold. He denied tobacco use. There was a family history of allergies, but the extent of these allergies is unknown.

At the emergency room, the patient presented with periocular, lip, and mild tongue angioedema. His respiratory rate was elevated at 20, his blood pressure was slightly hypotensive at 105/65, and slight tachycardia was present with a pulse of 110 beats per minute. The rest of his physical examination was unremarkable.

Serum-specific fluorescence enzyme immunoassay IgE testing confirmed sensitization to multiple finned fish species, including seasonal allergens (Table 1).

**Table 1:** Selected Allergen-specific IgE Results Note: Anything greater than 0.10 kUA/l is indicative of allergen-specific IgE sensitization.

Lab Test	Result
Allergen, Oyster IgE	< 0.10 kUA/l
Allergen, Clam IgE	< 0.10 kUA/l
Allergen, Lobster IgE	< 0.10 kUA/l
Allergen, Shrimp IgE	< 0.10 kUA/l
Allergen, Crab IgE	< 0.10 kUA/l
Allergen, Tilapia IgE	0.86 kUA/l
Allergen, Halibut IgE	0.58 kUA/l
Allergen, Mackerel IgE	0.30 kUA/l
Allergen, Trout IgE	0.57 kUA/l
Allergen, Salmon IgE	0.39 kUA/l
Allergen, Tuna IgE	0.18 kUA/l
Allergen, Fish Cod IgE	0.74 kUA/l
Allergen, Goosefoot, Lamb's quarter IgE	< 0.10 kUA/l
Allergen, White ash IgE	0.16 kUA/l
Allergen, Aspergillus fumigatus IgE	< 0.10 kUA/l
Allergen, Timothy Grass IgE	1.30 kUA/l
Allergen, Dog dander IgE	< 0.10 kUA/l
Allergen, Saltwort, Russian Thistle IgE	< 0.10 kUA/l
Allergen, Mugwort IgE	< 0.10 kUA/l
Allergen, Common Ragweed IgE	3.49 kUA/l
Allergen, Elm IgE	< 0.10 kUA/l
Allergen, Oak IgE	< 0.10 kUA/l
Allergen, Mountain juniper IgE	0.52 kUA/l
Allergen, Common silver birch IgE	< 0.10 kUA/l
Allergen, Alternaria alternata IgE	0.77 kUA/l

**Figure 1:**
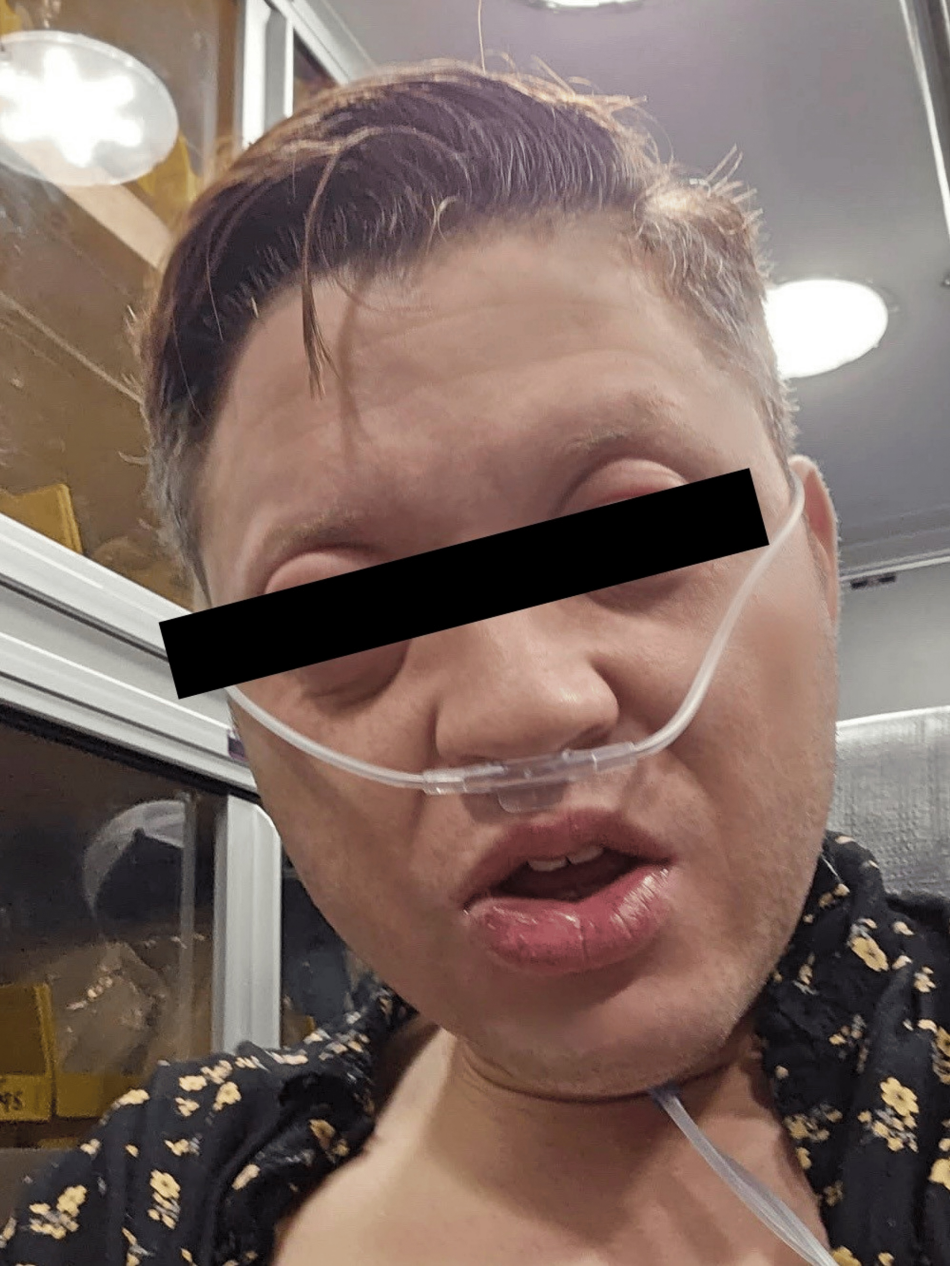
Physical manifestation of the reaction Note: Angioedema, throat tightness, and dyspnea noted. Informed written consent was obtained from the patient for the open-access publication of this case report as well as for the use of the above image.

Nine days after his initial reaction, the patient was referred to the Keesler Medical Center Allergy Clinic after severe reactivity to cod protein. He described his exposure as minimal, as he did not consume any fish-related protein, but instead ingested potatoes that had been placed in the proximity of cod fish. See Figure 1 for physical manifestation of his reaction.

Serum-specific lgE testing confirmed allergen-specific lgE sensitization to tilapia, halibut, mackerel, trout, salmon, tuna, fish cod, white ash, timothy grass, common ragweed, and mountain juniper (Table 1). After the serum-specific lgE testing was complete, further emphasis on avoidance of shellfish and finned fish was suggested. The patient was prescribed additional 0.3 mg epinephrine auto injectors, ordered to take Cetirizine 10 mg orally once or twice daily as needed for 12 months where he will then be reevaluated, and was educated on increased environmental safety by using proper air filters for aeroallergens. While there has been some research on oral challenges to increase tolerance, none have been verified yet.

## Discussion

This case illustrates a classic presentation of food-induced anaphylaxis in the setting of concurrent seasonal allergic rhinoconjunctivitis. Fish allergy is often persistent throughout adulthood, with cod and salmon among the most allergenic species [3]. The pathophysiology involves IgE-mediated mast cell degranulation upon exposure to fish parvalbumins or shellfish tropomyosin [3-6]. The patient’s seasonal allergic rhinoconjunctivitis is supported by his IgE profile and clinical symptoms. Grass, weed, and tree pollens are common allergens that trigger rhinitis symptoms in sensitized individuals. Airborne allergen exposure in occupational settings such as military bases may be exacerbated by fieldwork and training environments [5,7]. In individuals with a history of anaphylaxis, early epinephrine administration is the mainstay of treatment and has been shown to reduce both morbidity and mortality [2, 8]. Long-term management includes allergen avoidance, pharmacologic symptom control, and patient education on anaphylaxis recognition and response [8]. Food allergies, particularly to fish and shellfish, can have profound implications for individual safety and operational readiness in deployed environments where food selection is limited, and emergency medical services may be delayed or unavailable [1, 3, 5]. However, such a dramatic reaction, with low serum-specific IgE levels and minimal exposure, as was the case in this patient’s report, is unusual [6]. Anaphylaxis can be caused by IgE-independent variables such as direct mast cell activation, though as discussed, this is far less common [8]. Anaphylaxis poses a significant risk during military deployments, especially in austere or remote settings where heat or humidity may render epinephrine injections less effective. Unlike stateside environments, deployed locations may lack access to advanced medical care, and food labeling practices may not meet traditional U.S. Food and Drug Administration standards, increasing the risk of accidental allergen exposure [9, 10]. For service members with severe food allergies, like in this case, the requirement to carry an epinephrine auto-injector becomes not only a personal safety necessity but also a military command concern. Loss or damage of the auto-injector in the field, lack of proper storage facilities for medications, and difficulties in consistent allergen avoidance in dining facilities may hinder readiness for the military member [5, 10]. Additionally, environmental allergies can inhibit performance, alertness, and mission focus. Symptoms such as nasal congestion, sneezing, eye irritation, and fatigue during pollen seasons can impair sleep, concentration, and physical endurance, particularly in outdoor or field operations [4, 8]. For military personnel required to wear protective gear or gas masks, uncontrolled rhinitis symptoms can interfere with mask seal integrity or worsen discomfort, possibly compromising operational efficiency [5]. However, not all allergic individuals are deemed non-deployable. Instead, the Department of Defense evaluates an individual’s ability to deploy based on symptom control, risk mitigation strategies, and the ability of the service member to manage their condition in operational settings [5, 10]. In this case, the patient demonstrated good insight into his condition, adherence to allergen avoidance, and proper use of emergency medications. With continued education, risk management planning, and command awareness, he remains fit for duty without limitations from an allergy perspective. This case highlights the importance of pre-deployment medical screening, risk stratification, and contingency planning for allergic conditions. All service members with food-induced anaphylaxis should be equipped with epinephrine auto-injectors, detailed medical documentation, and mission-specific guidance for allergen exposure prevention. For environmental allergies, pharmacologic control and avoidance strategies can minimize symptom severity and ensure mission capability.

## Conclusions

This case displays the complexities that military readiness requirements have on otherwise manageable conditions. Severe reactivity with lower lgE to fish raises concerns for cross-contamination-induced anaphylaxis, especially in locations where traditional food testing is not available. Especially in certain contingency scenarios, the ability of military medical professionals to respond and properly treat an allergy-induced medical emergency can be diminished. However, while the reactivity with lower IgE to fish is of concern, with proper adherence and vigilance, this does not disqualify the member from deployments. Military service members with food-induced anaphylaxis and seasonal allergies benefit from a comprehensive evaluation, including allergen-specific IgE testing and environmental assessment. A tailored management approach enhances safety, symptom control, and operational readiness. Ensuring military members have the patient education required for management of their allergic diseases, as well as the proper medications prior to deploying, is vital to their safety in both controlled and austere environments.
